# Changes in sexual health following total hip arthroplasty in heterosexual patients with stiff hips and their partners: a retrospective study

**DOI:** 10.3389/fmed.2026.1671739

**Published:** 2026-02-26

**Authors:** Xiaojin Wu, Shiyong Wang, Rudong Chen, Haitao Hu, Rong Ma, Yanbin Tian, Zhaohui Ge

**Affiliations:** 1 Department of Orthopedics, General Hospital of Ningxia Medical University, Yinchuan, Ningxia, China; 2 The First Clinical Medical College, Ningxia Medical University, Yinchuan, Ningxia, China; 3 Yinchuan Guolong Orthopedic Hospital, Yinchuan, Ningxia, China

**Keywords:** heterosexuals, partners, sexual health, stiff hip, total hip arthroplasty

## Abstract

**Background:**

Sexual activity is an integral component of a healthy life. However, research on changes in sexual activity among patients with stiff hips and their partners before and after total hip arthroplasty (THA) remains limited. This study aims to investigate alterations in sexual activity following THA in heterosexual patients and their partners, and to assess its impact on sexual satisfaction, quality of life, and dyadic relationships.

**Methods:**

An anonymous, specifically designed sexual health survey was distributed to 65 patients undergoing THA for stiff or fused hips and their partners. This survey assessed changes in sexual satisfaction and activity, quality of life (QoL), and influencing factors from both patient and partner perspectives pre- and post-operatively. It also evaluated perioperative sexual health counseling received by patients. Each questionnaire item was analyzed independently using statistical software.

**Results:**

Forty eight patients provided analyzable data at study completion. Significant improvements (*p* < 0.001) were observed in VAS scores, Harris Scores, and hip range of motion (ROM) at final follow-up. The mean time to resume sexual activity post-THA was 3.6 ± 1.4 months (male 2.9 ± 1.2 vs. female 4.5 ± 1.1). By final follow-up, QoL and dyadic relationships showed marked improvement. Sexual frequency increased in 93.7% of patients, 60.4% adopted varied coital positions, and mean sexual satisfaction rose from 24% preoperatively to 82% postoperatively (*p* < 0.001). The primary factors limiting preoperative and postoperative sexual activity were restricted hip mobility, pain, and fear of prosthesis damage. No patients received perioperative sexual health counseling, yet 85.4% desired professional rehabilitation guidance, and 25% requested communication strategies with partners and pain management techniques. Regarding surgical decision-making, 60.4% would consider THA if hip pathology solely impaired sexual health. Partners reported significant improvements in patients’ QoL and sexual health postoperatively. Although partners expressed concerns about causing physical discomfort, all were willing to make adaptive adjustments.

**Conclusion:**

Stiff hips significantly impact patients’ sexual health. THA demonstrates positive effects on sexual activity, QoL, and dyadic relationships for both patients and their partners. It is therefore essential to enhance healthcare providers’ awareness of patients’ sexual needs and provide individualized perioperative sexual counseling.

## Introduction

Hip osteoarthritis (OA) is the leading cause of disability globally, with an estimated age-standardized prevalence of 417.7 per 100,000 people worldwide (1). It imposes a significant disease burden and demonstrates an increasing prevalence among younger populations (2–4). And with the recent COVID-19 pandemic, the incidence of hip joint vascular necrosis has increased dramatically, with more and more young patients undergoing surgery (5, 6). Existing studies have shown that two-thirds to four-fifths of patients with hip OA report sexual difficulties, primarily attributed to hip pain and restricted mobility (7–9). One-quarter of patients attribute marital dissatisfaction and interpersonal strain to their OA (10). As one of the most successful procedures globally, total hip arthroplasty (THA) enhances sexual satisfaction and function in the majority of patients (8, 11).

Sexual activity is recognized as a vital component of social well-being, distinct from mental and physical health, constituting part of overall health-related quality of life (HRQOL) (12). Since 1974, the World Health Organization (WHO) has advocated for sexual health, defining it as a state of physical, emotional, mental, and social well-being in relation to sexuality; it is not merely the absence of disease, dysfunction, or infirmity (12).

Sexual activity remains a sensitive and often stigmatized topic (13), yet it is closely linked to the well-being of couples and families. Research indicates a positive correlation between the severity of hip pathology and limitations in sexual activity (11). Current evidence confirms inadequate perioperative sexual health counseling. Dahm et al. (8) reported that 89% of patients desired more information regarding resuming sexual activity after THA. In a survey of sexual satisfaction in 101 patients after THA, Rougereau et al. (14) found that 96% stated their surgeon did not discuss sexual activity during preoperative consultations, while 77.5% desired postoperative guidance on this topic. In reality, communication between clinicians and patients on sexual health rarely occurs. Even among the minority of surgeons who address it, discussions typically last less than 5 min (8).

Previous studies have confirmed the significant positive impact of THA on postoperative sexual activity (11, 14, 15). Perioperative sexual education further enhances patient sexual satisfaction and QoL (16). Martino et al. (17) reported improved coital quality in 64% of patients post-THA, while 34% noted no change. In a prospective cohort study, Bonilla et al. (7) demonstrated an increase in female sexual satisfaction from 29% preoperatively to 93% postoperatively.

Current research primarily focuses on standard hip pathology with preserved mobility. To our knowledge, no studies have specifically investigated changes in sexual health and influencing factors following THA in patients with severely restricted hip mobility, namely stiff or fused hips. This study was therefore conducted to examine alterations in sexual health status before and after THA in this specific patient population. Furthermore, incorporating the partner perspective, it assesses postoperative recovery of sexual activity, satisfaction levels, and the impact on the dyadic relationship.

## Materials and methods

### Patient population

**Figure 1:**
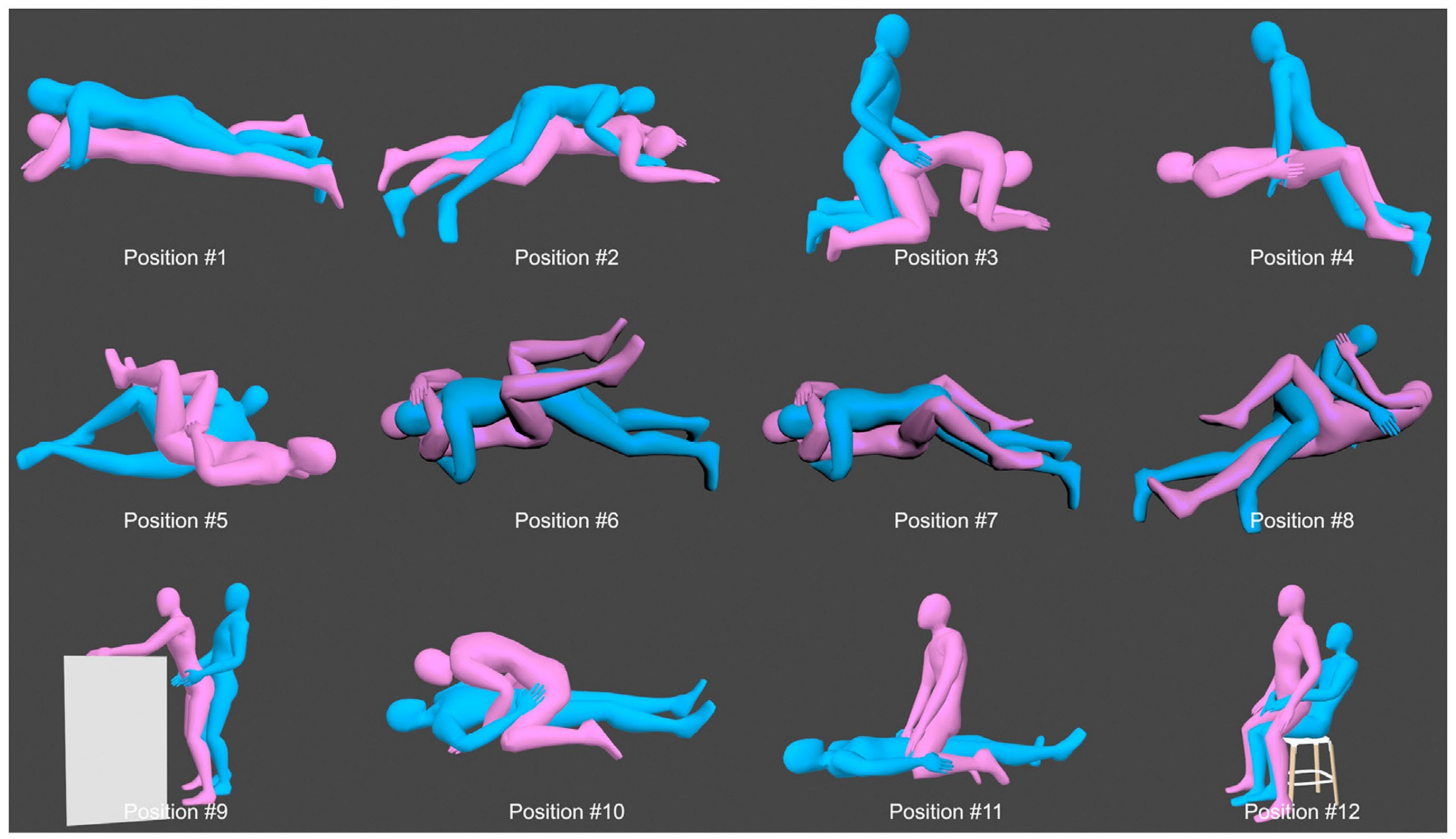
The 12 common sexual positions used in this study. The man is represented in blue and the woman in pink [Charbonnier et al. (31)].

Following approval by our Institutional Review Board, medical records of all patients undergoing THA for stiff or fused hips between January 2018 and December 2024 were reviewed. All procedures were performed via a posterolateral approach by one of two senior arthroplasty surgeons. Inclusion criteria were as follows:(1) Severe restriction of motion in the affected hip, i.e., a definite diagnosis of a stiff or fused hip; (2) at least 6 months after surgery; (3) between 25 and 60 years of age at the time of surgery; (4) signing of a written informed consent; and (5) Married or in a stable sexual relationship preoperatively. Exclusion criteria: (1) less than 6 months of follow-up; (2) History of ipsilateral hip trauma or surgery at other sites within the preceding 6 months; (3) Individuals who identified as homosexual or were in a same-sex relationship; (4) THA performed for other indications such as femoral neck fracture, periacetabular tumor, or preserved hip mobility; and (5) Comorbidities potentially affecting sexual function, such as cardiovascular, hepatic, and renal diseases, or endocrine disorders. The interval of 25–60 years of age at the time of surgery targeted the sexually active working-age population. The reason for the follow-up period of at least 6 months is that most people can resume sexual activity at 6 months postoperatively. For patients with bilateral stiff hips, only data following the second THA were collected to avoid confounding effects from the contralateral stiff hip after unilateral surgery. To minimize population heterogeneity and focus on the prespecified core variables, participation was restricted to individuals who self-identified as heterosexual and were in heterosexual relationships. This decision was driven by the fact that the sexual health questionnaire we designed and the referenced coital position diagrams (Figure 1) were specifically developed and validated for heterosexual couples. Therefore, to ensure consistency in data collection and interpretation, only heterosexual participants were included.

To protect privacy, patients could refuse to answer without disclosing their sexual status or willingness to participate. All participants were informed that their sensitive information would be kept absolutely confidential and that no personal information could be identified. Only the authors had access to the raw data and consent forms.

Following informed consent, eligible patients were given a self-developed sexual health questionnaire. This anonymous survey was collected by two opposite-sex observers not involved in clinical care, reporting only basic demographic data: age, gender, diagnosis, surgical date, and follow-up duration.

### Perioperative management

After admission and routine preoperative evaluation to exclude surgical contraindications, all patients underwent surgery performed by a senior arthroplasty surgeon via a posterolateral approach using non-cemented prostheses. A double osteotomy of the femoral neck was performed intraoperatively in all cases. For patients with bony hip fusion, acetabular reaming commenced directly after determining the normal anatomical position and optimal angle. In other patients, reaming proceeded using the true acetabulum as the anatomical landmark until the desired size was achieved. Bearing surface type and fixation method were determined individually by the surgeon based on patient characteristics, while acetabular cup size was selected based on optimal bone contact, coverage, and stability. Following implantation, the external rotators were repaired before wound closure, accompanied by local injection of an analgesic-anesthetic cocktail.

On the day of surgery, patients were allowed to stand and ambulate with physiotherapist guidance after full post-anesthesia recovery. During hospitalization, they received physical therapy focused on achieving independent gait, utilizing assistive devices under therapist supervision. Patients were also provided education on post-THA care, emphasizing return to daily activities, prevention of hip dislocation and periprosthetic infections. Following discharge, a prescribed home exercise program was implemented to walk without any external assistance as soon as possible.

### Sexual health questionnaire

Although several scales to assess sexual functioning are available, none specifically address sexual satisfaction related to hip dysfunction. To address this, we developed a 24-item face-to-face sexual health questionnaire comprising four modules: patient demographics, preoperative status, postoperative status, and partner perspectives. It focuses on time to sexual recovery, influencing factors, sexual satisfaction, QoL impact, perioperative counseling received/desired, and—critically—partner-reported postoperative sexual activity recovery and its effects on relationship dynamics and QoL. As the questionnaire consists of independent items, analyses were conducted per question rather than using composite scores (Questionnaire content: Appendix 1). To minimize embarrassment and ensure accuracy with sensitive topics, interviewers were gender-matched to participants.

### Data collection

Demographic data, clinical characteristics, and sexual health questionnaire responses were collected from patients and their partners. Demographic variables included: sex, age, marital status, diagnosis, disease duration, duration of sexual activity impairment, follow-up period, postoperative complications, and partner health status. Clinical characteristics comprised: pre−/postoperative VAS scores, ROM of the affected hip, Harris Hip Scores, and all 24 sexual health questionnaire items.

### Statistical analysis

SPSS 22.0 (IBM, Chicago, IL, USA) was used for data analysis. Quantitative data with normal distribution are represented by mean ± standard deviation (x̄ ± s), while categorical data are represented by frequencies and percentages (%). An independent samples *t*-test was used for intergroup comparisons of normally distributed data. The chi-square test was applied to assess associations of ordinal and categorical data, and the Bonferroni method was used to analyze differences across disease strata. The significance level was set at *α* = 0.05 (two-tailed), and *p* < 0.05 was considered statistically significant.

## Results

A total of 65 patients with stiff hips underwent THA. Ten patients without sexual partners and seven who declined the questionnaire were excluded, and finally 48 patients (22 females and 26 males) were enrolled in the study. These patients underwent a total of 53 THA procedures, including five who had bilateral surgery, with a mean inter-surgical interval of 1.8 ± 0.6 months. Mean age was 47.0 ± 9.7 years (range 25–60), with a mean follow-up of 26.2 ± 23.1 months. Diagnoses included ankylosing spondylitis (AS, *n* = 25), developmental dysplasia of the hip (DDH, *n* = 13), and OA (*n* = 10). Based on hip ROM, 12 were classified as fused hips and 36 as stiff hips. Except for one patient who experienced increased pain and reduced satisfaction due to sciatic nerve injury, no complications (e.g., periprosthetic infection, loosening, or dislocation) occurred during follow-up. All sexual partners of the patients were free from physical disorders and identified as heterosexuals. Patient demographics are shown in Table 1. Postoperatively, significant improvements were observed in VAS scores, Harris Hip Scores, and hip ROM (Table 2).

**Table 1:** Demographic variables of the study group.

Variable	N (48)	%
Gender	22 females	45.8%
	26 males	54.2%
Age (years)	47.0 ± 9.7	
Types of diseases	AS 25 cases	52.1%
	DDH 13 cases	27.1%
	OA 10 cases	20.8%
With/without ROM	Fused hip 12 cases	25%
	Stiff hip 36 cases	75%
Duration of hip disease (Years)	13 ± 8.9	
Duration of limited sexual activity (Years)	8.9 ± 5.8	
Follow up time (months)	26.2 ± 23.1	
Complications	Sciatic nerve injury 1 case	2.1%
Partner Health	Well	100%

ROM, range of motion; AS, ankylosing spondylitis; DDH, developmental dysplasia of the hip; OA: osteoarthritis.

**Table 2:** Outcomes of clinical characteristics.

Clinical characteristics	Preoperative	Final follow-up	T	*p*
Hip ROM (°)	32.43 ± 19.96	196.56 ± 22.00	−38.27	<0.001
Harris score	29.88 ± 10.32	90.60 ± 7.99	−32.24	<0.001
VAS score	6.50 ± 1.37	1.6 ± 1.27	18.19	<0.001

ROM, range of motion; VAS, Visual Analog Scale.

**Figure 2:**
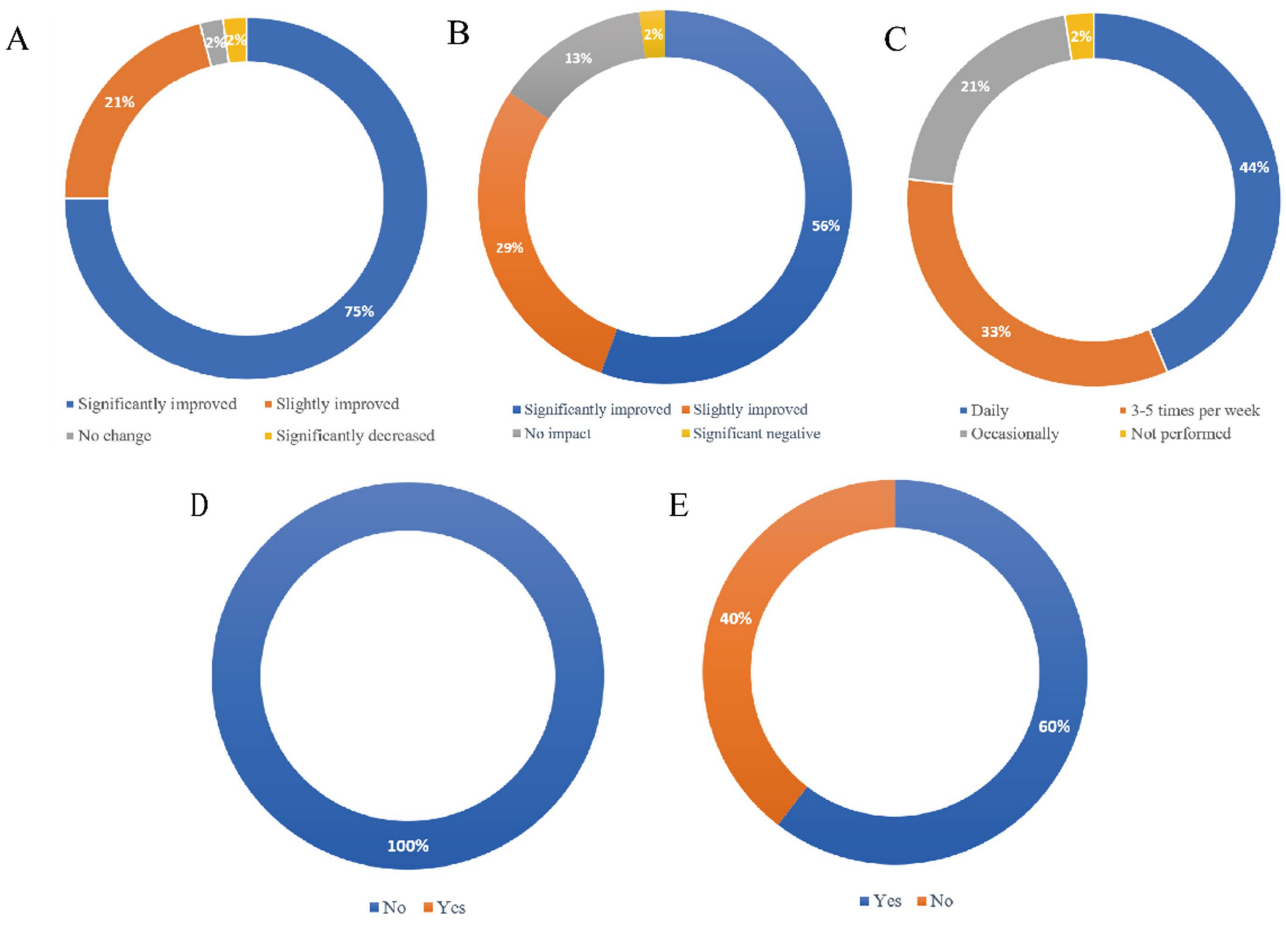
The results of partial independence analysis for patient sexual health: **(A)** The impact on quality of life; **(B)** the impact on the relationship dynamics; **(C)** postoperative rehabilitation frequency; **(D)** sexual health guidance; **(E)** solely impaired sexual, would surgery be considered.

Sexual health survey results indicated a mean disease duration of 13 ± 8.9 years. Preoperatively, all patients experienced hip disease-related sexual impairment for a mean duration of 8.9 ± 5.8 years. The primary limiting factor was restricted hip ROM, followed by pain, stiffness, and psychological factors. Sexual activity resumed at a mean of 3.6 ± 1.4 months postoperatively, a significantly earlier resumption time in men compared to women (M 2.9 ± 1.2 vs. F 4.5 ± 1.1, *p* < 0.001). At final follow-up, significant improvements were observed in overall sexual frequency (93.7% of patients) and satisfaction scores (preoperative: 2.4 ± 1.3 vs. postoperative: 8.2 ± 1.7). Two patients (4.2%) reported unchanged sexual activity, while one DDH patient (2.1%) experienced deterioration due to complications. In disease stratification analysis, although patients with AS reported lower preoperative satisfaction, all three diagnostic groups demonstrated significant postoperative improvement, with no statistically significant differences among them (Table 3). With the resumption of sexual activity, 95.8% of patients demonstrated QoL improvement. Postoperative sexual activity was primarily limited by: prosthetic concerns (loosening/dislocation), pain/stiffness, restricted motion, and psychological factors. A significant increase occurred in patients altering coital positions (preop 37.5% vs. postop 60.4%; *p* < 0.05). Chronic analgesic/biologic usage decreased substantially (93.7% preop vs. 31.3% postop), with all postoperative users having AS, and a statistically significant difference across disease subgroups. Regarding rehabilitation, 77.1% exercised daily or 3–5 times/week, while 20.8% exercised occasionally. No patients (0%) received perioperative sexual health counseling; however, 85.4% desired professional guidance, and 25% requested communication strategies with partners and pain management. Notably, 60.4% of patients (M:58.6%, F:41.4%) would consider THA if hip pathology solely impaired sexual health. Comparative sexual health outcomes between preoperative and final follow-up are presented in Table 4, with independently analyzed findings shown in Figure 2.

**Table 3:** Sexual health outcomes and associated factors in different diseases.

Items	Preoperative (*n*/x̄ ± s)					Final follow-up (*n*/x̄ ± s)				
	Variable	AS	DDH	OA	*p*	Variable	AS	DDH	OA	*p*
Medication usage (multiple choice)	NSAIDs	2 (8.0%)	6 (46.1%)	5 (50.0%)	<0.001^a^	NSAIDs	0 (0.0%)	0 (0%)	0 (0%)	0.01^a^
	Biologics	10 (40.0%)	0 (0.0%)	0 (0.0%)	0.001^b^	Biologics	0 (0.0%)	0 (0%)	0 (0%)	0.008^b^
	Corticosteroids	6 (24.0%)	0 (0.0%)	0 (0.0%)		Corticosteroids	0 (0.0%)	0 (0%)	0 (0%)	
	Analgesics	14 (56.0%)	8 (61.5%)	5 (50.0%)		Analgesics	14 (56.0%)	1 (7.7%)	0 (0%)	
	None	0 (0.0%)	1 (7.7%)	2 (20.0%)		None	11 (44.0%)	12 (92.3%)	10 (100.0%)	
Time to resumption of sexual activity							3.6±1.6	3,5±1.5	3.3±1.5	
Sexual frequency	1-3 times per month	8 (32.0%)	8 (61.5%)	7 (70.0%)		Significantly improved	18 (72.0%)	9 (69.2%)	8 (80.0%)	
	Less than once per month	13 (52.0%)	4 (30.8%)	3 (30.0%)		Slightly improved	5 (20.0%)	3 (23.1%)	2 (20.0%)	
	None	4 (16.0%)	1 (7.7%)	0 (0.0%)		No change	2 (8.0%)	0 (0.0%)	0 (0.0%)	
						Significantly decreased	0 (0.0%)	1 (7.7%)	0 (0.0%)	
sexual satisfaction		2.0±1.3	3.1±1.0	2.6±1.3	0.008^a^		8.2±1.4	7.8±2.4	8.6±1.3	
Changing sexual posture	Yes	8 (32.0%)	5 (38.5%)	5 (50.0%)		Yes	14 (56.0%)	7 (53.8%)	8 (80.0%)	
	No	17 (68.0%)	8 (61.5%)	5 (50.0%)		No	11 (44.0%)	6 (46.2%)	2 (20.0%)	
limiting factor (multiple choice)	Pain or stiffness	17 (68.0%)	7 (53.8%)	5 (50.0%)		Pain or stiffness	12 (48.0%)	4 (30.8%)	2 (20.0%)	
	Restricted hip ROM	24 (96.0%)	12 (92.3%)	9 (90.0%)		Restricted hip ROM	9 (36.0%)	2 (15.4%)	1 (10.0%)	
	Medication side effects	2 (8.0%)	1 (7.7%)	0 (0.0%)		Medication side effects	2 (8.0%)	0 (0.0%)	0 (0.0%)	
	Psychological stress	11 (44.0%)	6 (46.2%)	5 (50.0%)		Psychological stress	7 (28.0%)	1 (7.7%)	1 (10.0%)	
	Partner aspects	1 (4.0%)	1 (7.7%)	0 (0.0%)		Partner aspects	1 (4.0%)	1 (7.7%)	0 (0.0%)	
	Lack of medical guidance	1 (4.0%)	2 (15.4%)	1 (10.0%)		Lack of medical guidance	4 (16.0%)	2 (15.4%)	1 (10.0%)	
	None	1 (4.0%)	1 (7.7%)	1 (10.0%)		Fear prosthetic damage	19 (76.0%)	8 (61.5%)	5 (50.0%)	
						None	3 (12.0%)	2 (15.4%)	3 (30.0%)	
What support do you hope to receive						Rehabilitation guidance	23 (92.0%)	11 (84.6%)	7 (70.0%)	
						Psychological counseling	0 (0.0%)	0 (0.0%)	0 (0.0%)	
						Communication skills	6 (24.0%)	4 (30.8%)	2 (20.0%)	
						Pain management	9 (36.0%)	2 (15.4%)	1 (10.0%)	
						None	1 (4.0%)	2 (15.4%)	2 (20.0%)	

a Representing the statistical analysis results between AS and DDH.

b Representing the statistical analysis results between AS and OA.

**Table 4:** Patient sexual health outcomes and associated factors at preoperation and final follow-up.

Items	Preoperative		Final follow-up		*p*
	Variable	*n*/x̄ ± s	Variable	*n*/x̄ ± s	
Medication usage (multiple choice)	NSAIDs	13 (27.1%)	NSAIDs	0 (0%)	<0.001
	Biologics	10 (20.8%)	Biologics	0 (0%)	
	Corticosteroids	6 (12.5%)	Corticosteroids	0 (0%)	
	Analgesics	27 (56.3%)	Analgesics	15 (31.3%)	
	None	3 (6.3%)	None	33 (68.8%)	
Sexual frequency	1–3 times per month	23 (47.9%)	Significantly improved	35 (72.9%)	
	Less than once per month	20 (41.7%)	Slightly improved	10 (20.8%)	
	None	5 (10.4%)	No change	2 (4.2%)	
			Significantly decreased	1 (2.1%)	
Sexual satisfaction		2.4±1.3		8.2±1.7	<0.001
Changing sexual posture	Yes	18 (37.5%)	Yes	29 (60.4%)	0.02
	No	30 (62.5%)	No	19 (39.6%)	
limiting factor (multiple choice)	Pain or stiffness	29 (60.4%)	Pain or stiffness	18 (37.5%)	<0.001
	Restricted hip ROM	45 (93.7%)	Restricted hip ROM	12 (25.1%)	
	Medication side effects	3 (6.3%)	Medication side effects	2 (4.2%)	
	Psychological stress	22 (45.8%)	Psychological stress	9 (18.8%)	
	Partner aspects	2 (4.2%)	Partner aspects	2 (4.2%)	
	Lack of medical guidance	4 (8.3%)	Lack of medical guidance	7 (14.6%)	
	None	3 (6.3%)	Fear prosthetic damage	32 (66.7%)	
			None	8 (16.7%)	
What support do you hope to receive			Rehabilitation guidance	41 (85.4%)	
			Psychological counseling	0 (0.0%)	
			Communication skills	12 (25%)	
			Pain management	12 (25%)	
			None	5 (10.4%)	

ROM, range of motion; NSAIDs, non-steroidal anti-inflammatory drugs.

From partners’ perspectives, 93.8% reported improved sexual frequency postoperatively, with 81.3% noting significant improvement. Additionally, 89.6% perceived enhanced relationship dynamics due to sexual activity changes. Partner satisfaction scores averaged 8.1 ± 1.5 at final follow-up. The primary limiting factors were patients’ physical limitations (75.0%) and partners’ psychological stress (70.8%). Crucially, all partners expressed willingness to adapt regardless of circumstances (Table 5).

**Table 5:** Sexual health outcomes and associated factors from patient and partner perspectives at final follow-up.

Items	Patients		Partners		*p*
	Variable	*n*/x̄ ± s	Variable	*n*/x̄ ± s	
Sexual frequency	Significantly improved	35 (72.9%)	Significantly improved	39 (81.3%)	0.831
	Slightly improved	10 (20.8%)	Slightly improved	6 (12.5%)	
	No change	2 (4.2%)	No change	2 (4.2%)	
	Significantly decreased	1 (2.1%)	Significantly decreased	1 (2.1%)	
Sexual satisfaction		8.2±1.7		8.1±1.5	0.533
Limiting factor (multiple choice)	Pain or stiffness	18 (37.5%)	Patient’s physical limitations	36 (75.0%)	
	Restricted hip ROM	12 (25.1%)	Psychological stress	34 (70.8%)	
	Medication side effects	2 (4.2%)	Partner own health condition	0 (0.0%)	
	Psychological stress	9 (18.8%)	Insufficient communication	2 (4.2%)	
	Partner aspects	2 (4.2%)			
	Lack of medical guidance	7 (14.6%)			
	Fear prosthetic damage	32 (66.7%)			
	None	8 (16.7%)			
The impact on the relationship dynamics	Significantly improved	28 (58.3%)	Significantly improved	23 (47.9%)	0.598
	Slightly improved	13 (27.1%)	Slightly improved	20 (41.7%)	
	No impact	6 (12.5%)	No impact	4 (8.3%)	
	Significant negative	1 (2.1%)	Significant negative	1 (2.1%)	
Make adaptive adjustments			Very willing	32 (66.7%)	
			Willing	16 (33.3%)	

ROM, range of motion.

## Discussion

Sexual activity constitutes a vital component of adult quality of life, with hip mobility playing a fundamental role in its performance or engagement. Symptoms such as hip pain and limited mobility have been reported to start adversely affecting sexual activity approximately 2.5 years after symptom onset and may negatively impact partner relationships (14, 16). Consequently, restoring sexual capacity represents a critical postoperative objective (11, 18). Multiple pathologies, including AS-related hip disease, DDH, and hip OA, can progressively restrict hip mobility, potentially leading to stiff or fused hips. However, the severity of sexual impairment in such advanced cases remains undocumented. This retrospective study evaluated sexual activity changes before and after THA in patients with severe hip motion restriction. Results demonstrated significant postoperative improvements in hip ROM, VAS scores, and Harris Hip Scores among patients with stiff hips. Sexual satisfaction rates increased substantially from 24% preoperatively to 82% postoperatively, with 93.7% of patients reporting enhanced sexual frequency.

Preoperatively, all patients with stiff hips experienced compromised sexual function, with satisfaction rates averaging merely 24%—significantly lower than rates reported in the current literature. Contributing factors also differed from previous studies (7, 11, 13, 15, 19): whereas prior research identified pain as the primary limitation, restricted joint mobility predominated in our cohort, followed by pain. This discrepancy is likely attributable to our inclusion of patients with severe mobility limitations, including those with pain-free fused hips at the time of surgery. Postoperatively, patients reported concerns about prosthesis damage or dislocation during sexual activity, which is consistent with the existing literature (16, 20, 21). Actually, despite the fear of dislocation, only one case of dislocation during sexual activity has been reported to date (8), and the overall incidence has not been reported, but is estimated to be under 1% (22, 23).

Regarding medication use across disease types, although the number of patients on medication was significantly decreased at the final follow-up, persisting only in a subset of AS patients. Previous studies have indicated that both low back pain and the use of oral NSAIDs can adversely impact sexual health (19, 24–26), which may largely explain the lower preoperative satisfaction observed in AS patients. Postoperatively, although variations in satisfaction scores were noted, no statistically significant differences were found among the disease groups. Overall satisfaction improved from 24% preoperatively to 82% postoperatively; however, this rate appears modest compared with existing literature, where rates often exceed 90%. This discrepancy may be attributed not only to the influence of back pain and NSAID use in AS patients but also to the special patient population with stiff or fused hips, which represent more complex hip conditions than those typically examined in previous studies. Additionally, the prolonged duration of sexual limitations in our cohort may have exerted a greater impact on sexual health. Furthermore, there is a scarcity of studies focusing on sexual health in patients with complex hip disease or revision THA. While available literature suggests that revision THA, particularly with increasing numbers of surgeries and procedural complexity, negatively affects quality of life and hip function (27), our findings demonstrate that most patients with severe hip stiffness still achieve significant improvements in quality of life, hip function, and sexual health after primary THA. Therefore, an 82% satisfaction rate at final follow-up can be considered a clinically meaningful and positive outcome in this challenging patient population. Finally, aside from medication use and preoperative sexual satisfaction—which differed significantly in AS patients—no other questionnaire items showed statistically significant differences among disease groups postoperatively, despite overall improvements. Further large-sample or multi-center studies are warranted to validate these findings.

Although all patients in our survey reported experiencing some degree of negative impact on their sexual activity, only a small number experienced complete loss of sexual activity preoperatively. Notably, some respondents indicated that their sexual health might have been more significantly affected during the early stages of the disease. This observation aligns with findings by Östlund et al. (28), who suggested that patients with long-standing disease may develop strategies to adapt to their new situation over time. In our study, the average time from the onset of hip disease to the reported limitation in sexual activity was approximately 4 years. Given that this was a retrospective study, potential contributing factors to this prolonged duration may include adaptive behavioral adjustments between partners, in addition to recall bias among patients.

THA demonstrated a highly positive impact on sexual function in the current study. Consistent with previous studies (24, 29), the satisfaction, frequency of sexual activity and hip ROM were significantly improved postoperatively in our study. Critically, patients with stiff hips resumed sexual activity at a mean of 3.6 months, with females experiencing significantly delayed resumption compared to males—likely attributable to frequent use of positions requiring substantial hip abduction and external rotation (7, 14, 19). However, the overall recovery time lagged considerably behind the 6 weeks previously reported in the literature (13, 14, 18). In addition to the patients’ concern that the wound did not heal well and the lack of relevant health education during the perioperative period, the reason for this may also be related to the prolonged restriction of hip joint movement and its resultant wasting atrophy of periarticular muscles. Some studies (8) have also confirmed that patients with DDH have a relatively long time to resume sexual activity after surgery, which may be related to the healing of the surrounding soft tissues, and more time needs to be allowed for muscle healing. Generally, 1 month is the minimum time required for soft tissue healing and prevention of early dislocation (19). Furthermore, the surgical approach may influence the timing of resuming sexual activity; minimally invasive anterior and anterolateral approaches are considered less disruptive to periarticular structures and associated with a lower risk of early dislocation, thus permitting earlier resumption of sexual activity (17, 26). In contrast, all patients in this study were operated on via the posterolateral approach, which—combined with fear of dislocation leading to avoidance of certain positions—may have contributed to the delayed recovery. However, performing this procedure via an anterior approach can be challenging in patients with stiff or fused hips (30). Additionally, it has been suggested that the ultimate goal after THA is for the patient to be “unaware” of the prosthetic joint, and it is believed that the earlier the patient “forgets” about their new hip prosthesis, the sooner they can resume sexual activity (18). While literature recommends resuming sexual activity at 1–3 months (8, 19), our observations indicate 6–12 weeks post-THA is safe and permissible for stiff hip patients. Purposeful delay may enhance wound/periarticular tissue healing, thereby improving comfort and reducing instability risks (8).

Resumption of sexual activity post-THA significantly enhances QoL and partner relationships, aligning with established literature (15, 16, 26). Our findings demonstrate particularly pronounced effects: sexual activity improved QoL in 95.8% of patients, while 85.4% of patients and 89.6% of partners reported enhanced relationship dynamics. This heightened benefit profile may reflect our cohort’s relative youth, severe preoperative hip ROM, and consistent rehabilitation adherence.

Notably, coital positioning is largely influenced by hip ROM. All patients in our study presented with severe preoperative motion restriction. Postoperatively, as hip ROM improved and pain reduced, some patients attempted to modify their sexual positions, with a more pronounced change observed in males compared to females. Such gender differences have been frequently reported in previous studies (11, 13, 20). However, as sexual frequency and positioning behaviors evolved, proper coital techniques and dislocation avoidance emerged as primary concerns. All patients received standard perioperative education on dislocation risks and preventive strategies, such as avoiding extreme flexion, adduction, and internal rotation. Although none of our patients reported hip dislocation related to sexual activity during follow-up, earlier studies have raised concerns about motion-related risks. For instance, Charbonnier et al. (31) used motion capture technology and MRI to analyze hip joint movements in two prosthetic users during sexual intercourse. They found that female participants were more likely to perform motions involving flexion, abduction, and external rotation—movements associated with a higher risk of impingement compared to the rotational patterns typically seen in males (32). Combining 12 common sexual positions (Figure 1) used in the articles published by Charbonnier et al. (31) and Dahm et al. (8). Some researchers consider positions 1 and 9 to be the safest (22). However, Stegelmann et al. (23) employed CT-based robotic technology to evaluate high-risk coital positions. Their analysis accounting for acetabular cup orientation identified positions 2,3,4,6,7,9 and 12 as safe—demonstrating no prosthetic impingement in either gender. Moreover, they found that males exhibited higher impingement susceptibility than previously reported. Therefore, these findings suggest that dislocation risk assessments should incorporate acetabular cup orientation rather than relying solely on gender-based considerations. Concerning dislocation direction, males tend to experience anterior subluxation during intercourse, whereas females are more prone to posterior subluxation (19, 21). Synthesizing current evidence, Position 9 is considered the safest for all genders, whereas positions 5, 8, 10, and 11 should be particularly avoided.

Previous biomechanical and kinematic studies have identified critical hip angle thresholds for prosthetic impingement and instability (23, 31). For instance, females are more prone to impingement or dislocation with hip flexion >95° or abduction >32°, while males face higher risk with external rotation >40°. Notably, in males, impingement occurred in posture 8 across all acetabular cup orientations, most prominently at 30° of anteversion, whereas female patients exhibited greater susceptibility in posture 5. Importantly, even within the long-accepted “safe zone” (30°–50° inclination, 5°–25° anteversion), many sexual activity positions still cause impingement. Thus, postoperative sexual safety is best achieved by maintaining movement within a patient-specific biomechanical safety range—not by enforcing rigid positional restrictions. Furthermore, dual mobility implants mitigate prosthetic impingement more effectively without altering the incidence of bone impingement (23).

In addition to joint angles, movement patterns and activity intensity represent underappreciated biomechanical variables. Sexual activities characterized primarily by horizontal sliding involve relatively continuous load transfer and lower peak accelerations, whereas vertical thrusting may generate higher transient joint reaction forces and acceleration peaks. Given the limited direct *in vivo* measurements of joint loading during sexual activity following THA, we posit—based on biomechanical principles—that higher movement speed and intensity may elevate risks of implant micromotion, dislocation, and periprosthetic soft tissue strain, particularly during the early postoperative period when muscular and soft tissues have not fully recovered (32). Therefore, postoperative patient counseling should emphasize not only the timing for resuming sexual activity, but also controlled movement patterns, gradual intensity progression, and avoidance of high-risk combined motions. Integrating patient-specific implant positioning, restored ROM, and movement dynamics into such counseling can enhance patient confidence, mitigate unnecessary apprehension, and promote safer long-term functional recovery.

Another highlight of this study is the inclusion of partner perspectives on changes in sexual activity following THA. Partner responses demonstrated broad concordance with patient reports, with even more pronounced positive effects on relationship dynamics. Interestingly, partners identified patient physical limitations (75.0%) and their psychological stress (70.8%) as comparably prevalent barriers during intercourse, reflecting deep concern for the patient’s condition. When queried about adaptive adjustments for patients’ sexual activities, universal willingness was expressed—reinforcing established evidence on the critical role of partner communication in sexual rehabilitation (9).

Sexuality remains a sensitive topic for many individuals. Our findings indicate that patients did not receive any form of sexual health guidance during the perioperative period—neither did they seek advice from their physicians nor did physicians proactively provided information, which is a phenomenon previously reported in other studies (10, 20, 25). However, our survey revealed that 85.4% of patients desired professional rehabilitation guidance postoperatively, while 25% requested communication strategies with partners and pain management—needs that persisted post-surgery (20). Paradoxically, despite evidence suggesting that patients may benefit from understanding potential issues related to sexual activity (33), its implementation is hindered by concerns regarding privacy violations, discomfort, and cultural taboos (17, 21, 34). Additionally, the literature further indicates that healthcare professionals often omit sexual health from clinical assessments unless prompted by patients, potentially due to: unawareness of disease-related sexual impacts, perceived irrelevance (25), insufficient guideline familiarity, or personal discomfort when providing instructions (8). Furthermore, surgeons have been reported to be more likely to discuss sexual health recovery with married patients than with single individuals (8). Overall, however, the amount of sexual health information provided to patients by physicians remains very limited.

Given that sexual health constitutes a vital dimension of quality of life, the restoration of sexual function should be considered a critical postoperative goal. Consequently, the provision of sexual health counseling should be regarded as an essential competency for arthroplasty surgeons. While individual needs vary, healthcare providers should proactively create opportunities for sexual health discussions. We advocate for enhanced surgeon education in sexual medicine and the delivery of standardized patient counseling. Rehabilitation therapists serve as effective conduits for sexual health education (25)—integrating guidance during exercises, distributing materials, or facilitating discussions. Gender-concordant provider-patient dyads may optimize communication comfort (20). Digital platforms (e.g., instructional videos) represent viable alternatives to mitigate discomfort during sensitive conversations. Ultimately, patients should receive individualized protocols incorporating gender, age, radiographic implant positioning, soft-tissue recovery status, coital preferences, and sociocultural context (17, 21).

When queried whether they would consider THA if hip pathology solely affected sexual activity, 60.4% of patients responded affirmatively—a threefold increase compared to the 20% reported three decades ago (35). This shift reflects society’s growing recognition of sexual health’s importance and individuals’ increasing willingness to openly express and pursue it. Given this trend, after understanding the patient’s needs, motivations and expectations for sexual activity (20, 34), should sexual health be considered as a potential indication for THA in the future? This may present a challenging dilemma for surgeons. In young patients with high functional demands, THA improves pain, mobility, quality of life, and sexual function (36). Consequently, surgical indications continue expanding (37). However, 2.3% of patients would decline surgery if it potentially impairs their sexual activity (20). Our findings indicate that in managing complex hip conditions such as stiff or fused hips, clinicians should extend their focus beyond the joint pathology itself and proactively address patients’ sexual health early in the care process.

This study has several limitations. First, its retrospective design introduces recall bias regarding preoperative sexual health status. During face-to-face interviews, participants or partners may have overreported outcomes due to social desirability bias when addressing sensitive topics. Second, the questionnaire used in this study was self-designed based on the authors’ concerns and was not externally validated. Third, this study was limited to heterosexual patients, as the survey instrument and sexual position guides were designed and validated for this population. Consequently, the findings may not be generalizable to individuals in same-sex relationships or with diverse sexual orientations. Future studies should aim to develop inclusive tools that capture a broader spectrum of sexual behaviors and relationships. Finally, satisfaction and stress related to sexual health may be influenced by social factors and physical and mental health, which could not be assessed in the current study.

## Conclusion

Hip stiffness has a significant negative impact on patients’ sexual health. THA not only alleviates hip symptoms but also yields substantial improvements in sexual activity, QoL, and interpersonal dynamics for both patients and partners. Most patients resumed sexual activity by 3.6 months postoperatively. Dramatically, no patients received perioperative sexual health guidance. It is essential to enhance healthcare providers’ awareness of patients’ sexual needs and provide individualized perioperative sexual counseling.

## Data Availability

The original contributions presented in the study are included in the article/Supplementary material, further inquiries can be directed to the corresponding authors.
