# Supplementary material for: Changes in sexual health following total hip arthroplasty in heterosexual patients with stiff hips and their partners: a retrospective study
Source: Front Med (Lausanne). 2026 Feb 26;13:1671739. doi: 10.3389/fmed.2026.1671739 (PMC12979484; doi:10.3389/fmed.2026.1671739)
Supplement: Supplementary file 1 [file Supplementary_file_1.pdf]

Questionnaire on Sexual Health in Patients Before and After Surgery

(Anonymous completion. Information strictly confidential. Results for medical research only)

Date of Completion:____________

Basic Information

Sex: ☐ Male ☐ Female Ethnicity: _______

Current Age: _____ years;

Marital Status: ☐ Married ☐ Divorced/Widowed ☐ Single (With / Without Partner) _______

Duration of Hip Joint Limitation: _______ years; Comorbidities: _______

Total Hip Arthroplasty (THA) Date: _____ Y _____ M (Unilateral/Bilateral: ☐ Left ☐ Right)

Postoperative Complications (e.g., infection, dislocation, prosthetic loosening):

☐ No ☐ Yes (Please specify: ____________)

Your Partner's physical condition, presence of other diseases: ☐ No; ☐ Yes

Preoperative Information (7 Questions)

Did you require long-term use of the following medications before surgery? (Multiple selection possible):

☐ Non-Steroidal Anti-Inflammatory Drugs (NSAIDs)
☐ Biologics (e.g., TNF inhibitors)
☐ Corticosteroids
☐ Analgesics (e.g., opioids)
☐ None/Other: ____________

Your pre-operative sexual activity frequency:

☐ Daily
☐ 1-3 times per week
☐ 1-3 times per month
☐ Less than once per month
☐ None

Pre-operative sexual activity satisfaction (0-10, 0 = Extremely dissatisfied)

0 1 2 3 4 5 6 7 8 9 10

Before surgery, did your hip problem interfere with your sexual life?

☐ Yes ☐ No

How long was your sexual life negatively affected? _______ Years _______ Months.

Did you experience the following difficulties during sexual activities? (Multiple selection possible):

☐ Physical (hip) pain or stiffness
☐ Restricted hip movement (e.g., inability to flex/abduct hip)
☐ Medication side effects (fatigue, decreased libido, or lack of energy)
☐ Psychological stress (e.g., anxiety, low self-esteem, body image issues)

☐ Partner attitude, difficulty cooperating, or health status
☐ Lack of medical guidance (e.g., safety advice)
☐ None/Other: ____________

Before surgery, did you frequently change sexual positions?
☐ Yes ☐ No

Postoperative Information (12 Questions)
Do you currently require long-term use of the following medications? (Multiple selection possible):
☐ Non-Steroidal Anti-Inflammatory Drugs (NSAIDs)
☐ Biologics (e.g., TNF inhibitors)
☐ Corticosteroids
☐ Analgesics (e.g., opioids)
☐ None/Other: ____________

How long after surgery did you resume sexual activity? _______ Months

Change in sexual activity frequency post-operatively (compared to pre-op):
☐ Significantly increased
☐ Slightly increased
☐ No change
☐ Slightly decreased
☐ Significantly decreased

Post-operative sexual activity satisfaction (0-10, 0 = Extremely dissatisfied)
0 1 2 3 4 5 6 7 8 9 10

After surgery, do you frequently change sexual positions?
☐ Yes ☐ No

What do you consider the main factors affecting post-operative sexual activity? (Multiple selection possible):
☐ Physical (hip) pain or stiffness
☐ Restricted hip movement
☐ Medication side effects (fatigue, decreased libido, or lack of energy)
☐ Psychological stress (e.g., anxiety, low self-esteem, body image issues)
☐ Partner attitude, difficulty cooperating, or health status
☐ Lack of medical guidance (e.g., safety advice)
☐ Concern about prosthetic damage (e.g., dislocation, loosening)
☐ None/Other: ____________

Impact of post-operative sexual activity on your quality of life:
☐ Significantly improved

☐ Slightly improved
☐ No impact
☐ Slight negative impact
☐ Significant negative impact

Impact of changes in post-operative sexual ability on your relationship with your partner:
☐ Significantly improved relationship
☐ Slightly improved relationship
☐ No impact
☐ Slight negative impact
☐ Significant negative impact

Frequency of post-operative/current rehabilitation exercises:
☐ Daily ☐ 3-5 times per week ☐ Occasionally ☐ Not performed

Did you receive any sexual health guidance after surgery? (Multiple selection possible)
☐ Yes (Content: ☐ Position advice ☐ Pain management ☐ Psychological support ☐ Other: _______)
☐ No

What support would you like to improve your sexual life? (Multiple selection possible)
☐ Professional rehabilitation guidance
☐ Psychological counseling
☐ Partner communication skills
☐ Pain management plan
☐ None/Other: _______

If your disease *only* affected your sexual life, would you consider or choose hip replacement surgery?
☐ Yes ☐ No

Partner's Perspective (To be completed by partner, 5 Questions)
Have you noticed any change in the patient's sexual activity frequency after surgery?
☐ Significantly improved
☐ Slightly improved
☐ No change
☐ Slightly decreased
☐ Significantly decreased

Impact of changes in the patient's post-operative sexual ability on your relationship:
☐ Significantly improved relationship
☐ Slightly improved relationship
☐ No impact
☐ Slight negative impact

☐ Significant negative impact

What do you consider the main factors affecting your sexual activity as a partner? (Multiple selection possible):

☐ Patient's physical limitations
☐ Psychological stress (e.g., worrying about patient discomfort)
☐ Limitations due to your own health condition
☐ Insufficient communication
☐ None/Other: ____________

Your satisfaction with current sexual activity (0-10, 0 = Extremely dissatisfied)

0 1 2 3 4 5 6 7 8 9 10

Are you willing to cooperate with the patient on adaptive adjustments (e.g., changing positions)?

☐ Very willing ☐ Willing ☐ Neutral ☐ Unwilling
